# Plasma metabolomic profiling reveals factors associated with dose-adjusted trough concentration of tacrolimus in liver transplant recipients

**DOI:** 10.3389/fphar.2022.1045843

**Published:** 2022-10-31

**Authors:** Huaijun Zhu, Min Wang, Xiaofu Xiong, Yao Du, Danying Li, Zhou Wang, Weihong Ge, Yizhun Zhu

**Affiliations:** ^1^ Department of Pharmacology, School of Pharmacy, Fudan University, Shanghai, China; ^2^ Department of Pharmacy, the Affiliated Drum Tower Hospital of Nanjing University Medical School, Nanjing, China; ^3^ Nanjing Medical Center for Clinical Pharmacy, Nanjing, China; ^4^ Department of Pharmacy, The First Affiliated Hospital of Zhengzhou University, Zhengzhou, China; ^5^ State Key Laboratory of Quality Research in Chinese Medicine and School of Pharmacy, Macau University of Science and Technology, Macau, China

**Keywords:** liver transplantation, tacrolimus, metabolomics, therapeutic drug monitoring, trough concentration

## Abstract

Inter- and intrapatient variability of tacrolimus exposure is a vital prognostic risk factor for the clinical outcome of liver transplantation. New factors or biomarkers characterizing tacrolimus disposition is essential for optimal dose prediction in recipients of liver transplant. The aim of the study was to identify potential plasma metabolites associated with the dose-adjusted trough concentration of tacrolimus in liver transplant recipients by using a global metabolomic approach. A total of 693 plasma samples were collected from 137 liver transplant recipients receiving tacrolimus and regular therapeutic drug monitoring. Untargeted metabolomic analysis was performed by ultraperformance liquid chromatography-quadrupole time-of-flight mass spectrometry. Univariate and multivariate analyses with a mixed linear model were conducted, and the results showed that the dose-adjusted tacrolimus trough concentration was associated with 31 endogenous metabolites, including medium- and long-chain acylcarnitines such as stearoylcarnitine (*β* = 0.222, *p* = 0.001), microbiota-derived uremic retention solutes such as indolelactic acid (*β* = 0.194, *p* = 0.007), bile acids such as taurohyodeoxycholic acid (*β* = −0.056, *p* = 0.002), and steroid hormones such as testosterone (*β* = 0.099, *p* = 0.001). A multiple linear mixed model including 11 metabolites and clinical information was established with a suitable predictive performance (correlation coefficient based on fixed effects = 0.64 and correlation coefficient based on fixed and random effects = 0.78). These data demonstrated that microbiota-derived uremic retention solutes, bile acids, steroid hormones, and medium- and long-chain acylcarnitines were the main metabolites associated with the dose-adjusted trough concentration of tacrolimus in liver transplant recipients.

## 1 Introduction

Tacrolimus is considered the primary immunosuppressant in solid organ transplantation and has been used in liver transplant recipients for approximately two decades (Muduma et al., 2016). Pharmacokinetics-guided dosing is recommended to individualize tacrolimus treatment due to the narrow therapeutic window and wide interindividual pharmacokinetic variability (Brunet et al., 2019). Although the dose-interval area under the curve (AUC) of tacrolimus would be best associated with clinical effects, a multiple sampling strategy is not feasible in clinical practice. A limited sampling strategy with trough concentration (C0) is regularly adopted in most center for therapeutic drug monitoring (TDM) (Brunet et al., 2019; Birdwell et al., 2015). Pharmacogenomics is also essential in pharmacokinetics of tacrolimus, with *CYP3A5* genotypes used to guide initial tacrolimus dosing (Brunet et al., 2019). Nevertheless, limitations and challenges for tacrolimus use exist. In liver transplantation, guidelines for *CYP3A5* genotype and tacrolimus dosing are recommended only when the donor and recipient genotypes are identical (Birdwell et al., 2015). Furthermore, genotypes provide less information and low predictive value for intrapatient variability (IPV) (Shuker et al., 2015), whereas a recent study identified IPV of tacrolimus exposure as a crucial prognostic risk factor for the clinical outcome in solid organ transplantation, including liver transplantation (Del Bello et al., 2018). Although the most important clinical cause influencing such IPV is medication nonadherence, which is modifiable by clinical interventions, other factors, including drug-drug interactions, food intake, herbal or nutritional constituents and gastrointestinal disorders, also have shown significant roles (Gonzales et al., 2020). Pathophysiological changes such as graft function recovery, inflammation, and altered plasma protein concentrations may affect the tacrolimus disposition of recipients after liver transplantation (Ganesh et al., 2017). Thus, the identification of novel factors or biomarkers characterizing tacrolimus disposition is essential for predicting the optimal dose in liver transplant recipients.

Cytochrome P450 3A isoenzymes (CYP3A), mainly CYP3A4 and CYP3A5, are involved in tacrolimus metabolism. Several human studies have been conducted to identify endogenous biomarkers of CYP3A activity. With the advent of serendipity and hypothesis-driven processes, urinary and plasma metabolites were evaluated as endogenous metrics through the main phenotyping validation criteria (Magliocco et al., 2019). The ability to provide insights into phenotypic metabolite changes with wide coverage and high throughput allows metabolomics to be a promising method for biomarker discovery (Tolstikov et al., 2020). Mass spectrum (MS)-based metabolomic methods involving targeted or untargeted approaches have also been used to detect novel CYP3A biomarkers. Urinary cortisol/6β-hydroxycortisol, cortisone/6β-hydroxycortisone, dehydroepiandrosterone (DHEA)/(7β-OH-DHEA plus 16α-OH-DHEA), and plasma cholesterol/4β-hydroxycholesterol are reliable predictive markers of hepatic CYP3A activity (Shin et al., 2013). Phapale et al. conducted a pharmacometabolomic study on the urine of healthy volunteers to identify a predictive metabolic phenotype of individualized tacrolimus pharmacokinetics and identified four metabolites, namely, cortisol, methylguanosine, acetyl-arginine, and phosphoethanolamine, representing steroid-related, nucleotide/purine, amino acid-related, and glycerophospholipid metabolism, respectively, to predict the pharmacokinetic parameters of tacrolimus (Phapale et al., 2010).

With respect to liver transplant recipients, many clinical factors and drugs could alter the metabolomic profile (Oweira et al., 2018). Thus, the biomarkers for tacrolimus disposition identified in healthy controls should be applied in clinical practice with caution, and a study performed in liver transplant recipients may provide more insight views for metabolomics-guided dosing. Additionally, dose-adjusted tacrolimus trough concentration (C0/D) was commonly used as a surrogate marker for dose response or an index of clearance in most of the studies conducted in patient (Riva et al., 2019). Thus, the present study attempted to identify potential plasma endogenous metabolites associated with tacrolimus C0/D in liver transplant recipients by using a global metabolomic approach.

## 2 Materials and method

### 2.1 Subjects

**FIGURE 1:**
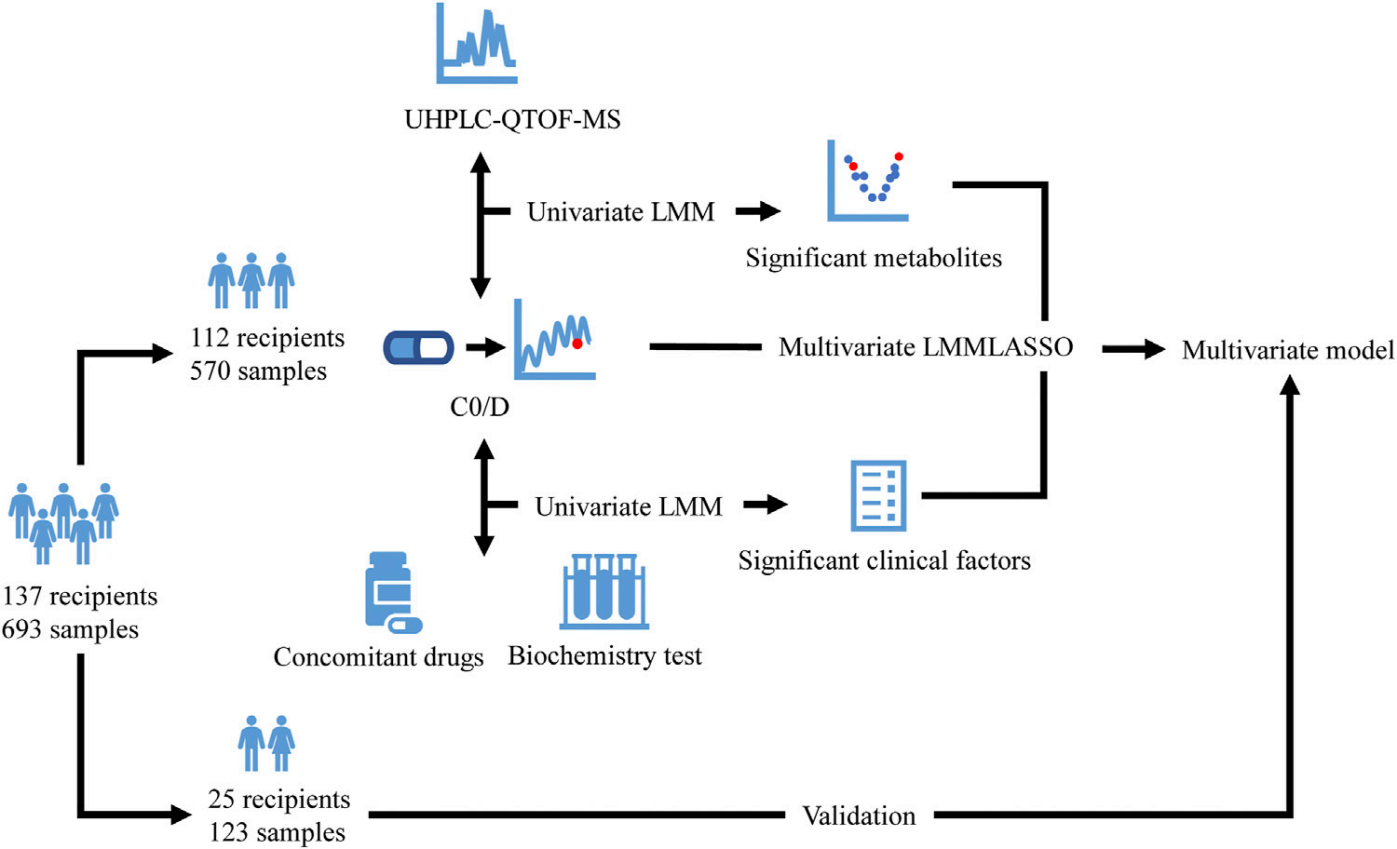
Flow diagram of the study. The enrolled recipients were divided into the model development group (112 recipients and 570 samples) and a validation group (25 recipients and 123 samples). The model development group data were used to identify potential metabolites detected by UHPLC-QTOF-MS and clinical factors associated with C0/D and to construct the multivariate model using LMM with LASSO, whereas validation group data were used to evaluate the model. UHPLC-QTOF-MS: ultrahigh-performance liquid chromatography-quadrupole time-of-flight mass spectrometry; C0/D: dose-adjusted tacrolimus trough concentration; LMM: linear mixed model; LASSO: least absolute shrinkage and selection operator.

The present retrospective study was conducted at the Hepatobiliary Center, the Affiliated Drum Tower Hospital of Nanjing University Medical School. Adult patients receiving liver transplantation between January 2018 and July 2019 were included. All the recipients received tacrolimus-based regimens and TDM. Subjects with incomplete electronic medical records, and patients undergoing multivisceral transplantation or other additional organ transplantation were excluded. Plasma samples for metabolomics study were available residual biosamples for TDM assay from the biobank of our laboratory. Samples with a C0 of tacrolimus lower than the quantitation limit or with a volume not enough for such analysis were excluded. The enrolled subjects were randomly divided into the model development group and a validation group. The model development group data were used to identify potential metabolites associated with C0/D and to construct the multivariate model, whereas validation group data were used to evaluate the results (Figure 1). The study was carried out in accordance with the principles of the Helsinki Declaration and approved by the Institutional Review Board at Nanjing Drum Tower Hospital (No. 2020-053-01). Informed consent was waived due to the deidentified data provided to researchers and residual biosamples used.

### 2.2 Treatment and TDM

Recipients received a tacrolimus-based immunosuppressive regimen including mycophenolate mofetil and corticosteroids. The initial dose of tacrolimus (Prograf^®^, Astellas, Killorglin, Ireland; or Saifukai^®^, Huadong Medicine, Hangzhou, China) was generally 2-3 mg orally twice daily, whereas the posttransplantation C0 target levels were 8–12 ng/ml in the first month, 7–10 ng/ml during the next months, and 5–7 ng/ml after the first year. TDM was performed for patients receiving tacrolimus at a fixed dose for about 3 days, and whole blood samples for C0 detection were taken within 1 hour before the morning dose. The concentration of tacrolimus was determined by the enzyme multiplied immunoassay technique (EMIT) using a drug testing system (Viva-E, Siemens Healthcare Diagnostics Inc, Erlangen, Germany) and EMIT^®^ 2000 tacrolimus assay kit (Siemens Healthcare Diagnostics Inc, Erlangen, Germany). The procedures were conducted according to the manufacturer’s instructions. The calibration range of the assay was 2.0–30 ng/ml, and samples with a C0 level lower than 2.0 ng/ml were excluded from the study. The performance of the tacrolimus assay in our laboratory was verified in an external quality assessment organized by the National Center for Clinical Laboratories. The internal quality assessment during the study was regularly performed, while the average bias was 6.47%, −1.67% and 1.35%, and the average coefficient of variation was 13.44%, 7.69% and 6.30% for low-, medium- and high-quality controls, respectively. An aliquot of 500 μL of whole blood was also centrifuged at 2,500 g for 10 min at 4°C once the sample was received in the clinical laboratory. The plasma and blood cells were stored at −80°C for further analysis.

### 2.3 Data collection

Patient data around C0 sampling were collected retrospectively from the hospital information system (HIS) and included daily doses of tacrolimus, weight, concomitant drugs (voriconazole, fluconazole, Wuzhi capsule, omeprazole or esomeprazole, caspofungin, and dihydropyridine calcium channel blockers [DHP CCB]), and blood biochemistry parameters. Wuzhi capsule is a Chinese patent medicine, and its abundant active ingredients include deoxyschizandrin, schisantherin A, schisandrol B and schisandrin (Qin et al., 2014). This is a common drug used to increase the exposure to tacrolimus in most transplantation centers in China.

### 2.4 Untargeted metabolomics

Plasma samples were thawed on ice, and a 40-μL aliquot was mixed with 160 μL of cold acetonitrile by vortexing for 5 min. After centrifugation at 20,000 g for 15 min at 4°C, the supernatant was collected and centrifuged again. The new supernatant was prepared for injection and separation using ultrahigh-performance liquid chromatography (UHPLC) (ExionLC AD UHPLC, ABSciex, Framingham, MA, United States) with a Kinetex C18 (2.6 µm, 100 mm × 2.1 mm, Phenomenex, CA, United States) column at 40°C. All samples were analyzed using a TripleTOF 5,600 + system (ABSciex, Framingham, MA, United States) in positive and negative electrospray ionization modes. Untargeted metabolomics methods and metabolomics data analysis are shown in the Supplementary Material.

### 2.5 Statistical analyses

The C0/D of tacrolimus was calculated by dividing the trough by the daily dose (D) normalized to actual body weight, whereas the log-transformed C0/D (log_2_C0/D) was investigated as the dependent variable in the subsequent univariate and multivariate analyses. The enrolled subjects were randomly divided into the model development group and a validation group using the function of “createDataPartition” in “caret” package in R software, and unsupervised principal component analysis (PCA) was adopted to evaluate the assay quality and the homogeneity of the metabolic data by using the SIMCA 14.1 (Umetrics, Uppsala, Sweden) software package. Considering the log_2_C0/D variation at two levels (between subjects and within subjects due to the repeated measures), metabolites associated with log_2_C0/D were identified using a linear mixed model, with each feature as the fixed effect and subject as the random effect on log_2_C0/D. *p* values of the fixed effect were adjusted by the false discovery rate (FDR) (“qvalue” package in R software) for multiple comparisons, and power was estimated by a simulation-based method (“simr” package in R software). Mummichog algorithm prediction (Li et al., 2013) was performed to identify enriched pathways.

To build a multivariate model in the model development group, all the metabolites and other factors including demographic information, biochemistry parameters and concomitant drug use with statistically significance in univariate analysis were included, and variable selection and penalization were performed by minimizing Akaike information criteria (AIC) using the method of least absolute shrinkage and selection operator (LASSO) augmented with 10-fold cross-validation. The predictive accuracy of the final model was assessed numerically in the model development and validation group through calculation of the correlation coefficient (R) by the fixed effects and full model, the mean error (ME), the mean absolute error (MAE), the mean relative error (MRE), and the relative root mean squared error (RMSE).

All statistical analyses were performed using STATA MP 16.0 (StataCorp, TX, United States), RStudio 1.3.959 (R Foundation for Statistical Computing, Vienna, Austria), and R-3.6.3 (R Foundation for Statistical Computing, Vienna, Austria). *p* values were two-sided, and values <0.05 were considered statistically significant.

## 3 Results

### 3.1 Subjects

A total of 137 liver transplant recipients and 693 samples were finally include in the study (Supplementary Figure S1). The median age of the patients was 49 years [interquartile range (IQR): 43–57 years], with a body mass index (BMI) of 23.31 (20.64–25.25) kg/m^2^. Most of the recipients were primarily diagnosed with end-stage liver cirrhosis (56.9%) and malignancies (19.7%) and underwent transplantation from a deceased donor (95.6%). Data regarding the concomitant medications were retrieved from HIS. Voriconazole was observed in 22 samples from eight patients, fluconazole in 65 samples from 17 patients, omeprazole or esomeprazole in 68 samples from 43 patients, and DHP CCB in 50 samples from 24 patients. Additionally, Wuzhi capsule was concomitantly administered in 198 samples from 85 subjects. The differences in the demographic information, clinical biochemistry parameters, and tacrolimus dose and concentration between the two groups were statistically nonsignificant (Table 1).

**TABLE 1:** Demographic and medical information of liver allograft recipients^a^.

Characteristics	Total subjects	Model development group	Validation group	*p* Value
Number of subjects (n)	137	112	25	
Age (years)	49 (43–57)	49 (42–56)	50 (43–58)	0.781
Sex (n)				
Female	35 (25.55%)	28 (25.00%)	7 (28.00%)	
Male	102 (74.45%)	84 (75.00%)	18 (72.00%)	0.753
Weight (kg)	65 (56–73.5)	63.75 (56–72.5)	68 (55–75)	0.628
Height (cm)	169 (163–173)	169 (163–173)	169 (161–171)	0.414
BMI (kg/m^2^)	23.31 (20.64–25.25)	23.03 (20.69–25.01)	24.08 (20.40–25.95)	0.444
History of smoking (n)	15 (10.95%)	11 (9.82%)	4 (16.00%)	0.371
History of alcohol consumption (n)	15 (10.95%)	11 (9.82%)	4 (16.00%)	0.371
Hypertension (n)	21 (15.33%)	17 (15.18%)	3 (12.00%)	0.684
Diabetes (n)	16 (11.68%)	15 (13.51%)	1 (4.00%)	0.182
Graft type (n)				
Deceased	131 (95.62%)	106 (94.64%)	25 (100.00%)	
Living	6 (4.38%)	6 (5.36%)	0 (0.00%)	0.237
Primary diagnosis (n)				
End-stage liver cirrhosis	78 (56.93%)	62 (55.36%)	16 (64.00%)	
Malignancies	27 (19.71%)	22 (19.64%)	5 (20.00%)	
Acute liver failure	19 (13.87%)	17 (15.18%)	2 (8.00%)	
Cholestatic liver disease	11 (8.03%)	9 (8.04%)	2 (8.00%)	
Metabolic liver disease	2 (1.46%)	2 (1.79%)	0 (0.00%)	0.905
Concomitant medication (n)				
Voriconazole	8 (5.84%)	6 (5.36%)	2 (8.00%)	0.572
Fluconazole	17 (12.41%)	15 (13.39%)	2 (8.00%)	0.460
Wuzhi capsule	85 (62.04)	66 (58.93%)	19 (76%)	0.112
Omeprazole/Esomeprazole	43 (31.39%)	35 (31.25%)	8 (32.00%)	0.942
Caspofungin	41 (29.93%)	32 (28.57%)	9 (36.00%)	0.463
DHP CBB	24 (17.52%)	22 (19.64)	2 (8.00%)	0.166
Warfarin	10 (7.30%)	10 (8.93%)	0 (0.00%)	0.208
Mycophenolic acid/Mycophenolate mofetil	129 (94.16%)	104 (92.86%)	25 (100%)	0.350
Magnesium isoglycyrrhizinate	99 (72.26%)	79 (70.54%)	20 (80%)	0.339
Clinical biochemistry parameters				
ALT (IU/L)	47.02 (22.55–91.03)	44.95 (22.35–89.03)	50.67 (26.28–121.08)	0.493
AST (IU/L)	34.53 (24.04–57.70)	32.85 (23.04–54.97)	41.34 (31.69–76.72)	0.077
GGT (IU/L)	61.80 (42.13–105.60)	63.87 (41.27–105.60)	60.84 (50.25–96.90)	0.787
Total bilirubin (μmol/L)	22.57 (12.92–37.43)	22.53 (12.65–37.50)	28.45 (15.87–35.05)	0.607
Direct bilirubin (μmol/L)	10.57 (5.23–23.77)	10.30 (4.93–24.70)	13.42 (7.03–23.36)	0.486
Albumin (g/L)	40.70 (38.83–42.97)	40.73 (38.87–42.97)	40.59 (38.67–42.97)	0.902
Glucose (mmol/L)	6.08 (5.34–7.64)	6.08 (5.28–7.64)	6.16 (5.55–7.06)	0.483
Creatinine (μmol/L)	67.82 (58.22–94.17)	67.00 (58.22–90.33)	71.50 (58.20–101.33)	0.444
Cholesterol (mmol/L)	3.18 (2.70–3.70)	3.19 (2.70–0.68)	3.15 (2.55–3.77)	0.670
Hematocrit (%)	34.40 (30.35–38.17)	34.40 (30.36–37.68)	33.74 (29.25–40.58)	0.834
Tacrolimus				
Total number of samples (N)	693	570	123	
Number of samples per patients	4 (3-6)	4 (3-6)	3 (3-5)	0.528
C0 (ng/ml)	7.50 (6.35–9.53)	7.42 (6.41–9.56)	8.03 (6.32–9.25)	0.732
Sampling time point (day after surgery)	89 (51.67–150.4)	92.81 (52.33–153.67)	78.25 (48.67–118.00)	0.311
Daily dose (mg)	3.16 (2.50–3.73)	3.15 (2.50–3.79)	3.25 (2.67–3.67)	0.776
Daily dose normalized (mg/kg)	0.048 (0.036–0.060)	0.048 (0.036–0.060)	0.045 (0.039–0.060)	0.843
C0/D [(ng/ml)/(mg/kg)]	178.08 (129.85–244.58)	180.48 (128.04–244.46)	170.33 (140.83–251.21)	0.585
log_2_ (C0/D) {log_2_ [(ng/ml)/(mg/kg)]}	7.33 (6.90–7.86)	7.31 (6.90–7.86)	7.34 (6.92–7.82)	0.734

^a^ Continuous variables are presented as median (25th–75th percentiles), whereas categorical variables are expressed as frequency and percentage. The Mann-Whitney *U* test and chi-square test (or Fisher’s exact test as appropriate) were used to compare the continuous and categorical data, respectively between the model development and validate groups. BMI: Body Mass Index; AST: aspartate aminotransferase; ALT: alanine aminotransferase; GGT: gamma-glutamyl transpeptidase; C0: trough concentration; C0/D: trough concentration normalized by daily dose (normalized by weight); DHP CCB: dihydropyridine calcium channel blockers.

### 3.2 Associations of demographic information and biochemistry parameters with log_2_C0/D in model development group

**FIGURE 2:**
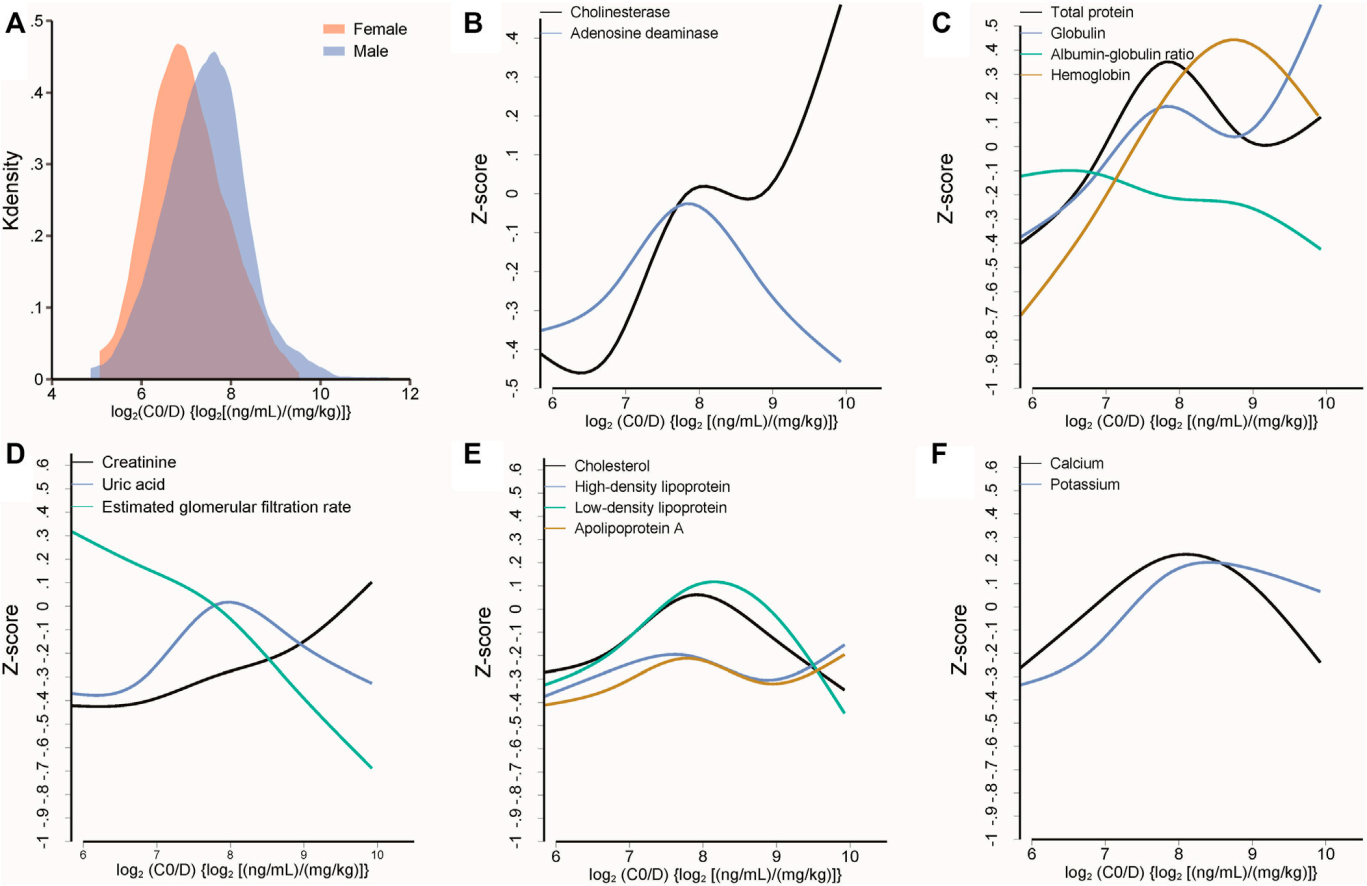
**(A)** Different distributions of log_2_C0/D of tacrolimus between female and male subjects. **(B–F)** Median spline plots of Z score of biochemical parameters *versus* log_2_C0/D of tacrolimus. All the plotted parameters were identified to be significantly associated with the log_2_C0/D of tacrolimus by a linear mixed model.

The linear mixed model exhibited that log_2_C0/D increased with postoperative days [*β* = 0.002, standard error (SE) = 0.0003, *p* < 0.001] and height (*β* = 0.026, SE = 0.009, *p* = 0.005). Male subjects exhibited a higher log_2_C0/D than females (*β* = 0.432, SE = 0.138, *p* = 0.002) (Figure 2 **. A**). Cholinesterase, adenosine deaminase, total protein, globulin, and hemoglobin were positively associated with log_2_C0/D. Tacrolimus pharmacokinetics were negatively associated with renal function and positively associated with parameters related to lipids (Figures 2B–F).

### 3.3 Metabolites identification by untargeted metabolomics analysis

The features were subjected to PCA after data normalization, and the results are illustrated in Supplementary Figures S2A,B. Quality control samples were clustered well in both ion modes, indicating platform stability and metabolomics data reliability. The illustration of grouping homogeneity suggested no evident separation trends between the model development and validation groups in either mode. Pearson’s correlation analysis exhibited good agreement of creatinine, urea, and uric acid levels between the levels obtained in the clinical laboratory and intensity in the metabolomics data (creatinine: R = 0.95, *p* < 0.001; urea: R = 0.94, *p* < 0.001; uric acid: R = 0.90, *p* < 0.001; Supplementary Figure S2C). Obvious differences were also observed for voriconazole, fluconazole, and omeprazole at the metabolomics level between samples with and without drug use, indicating high reliability of the metabolomics data (Supplementary Figure S2D).

**FIGURE 3:**
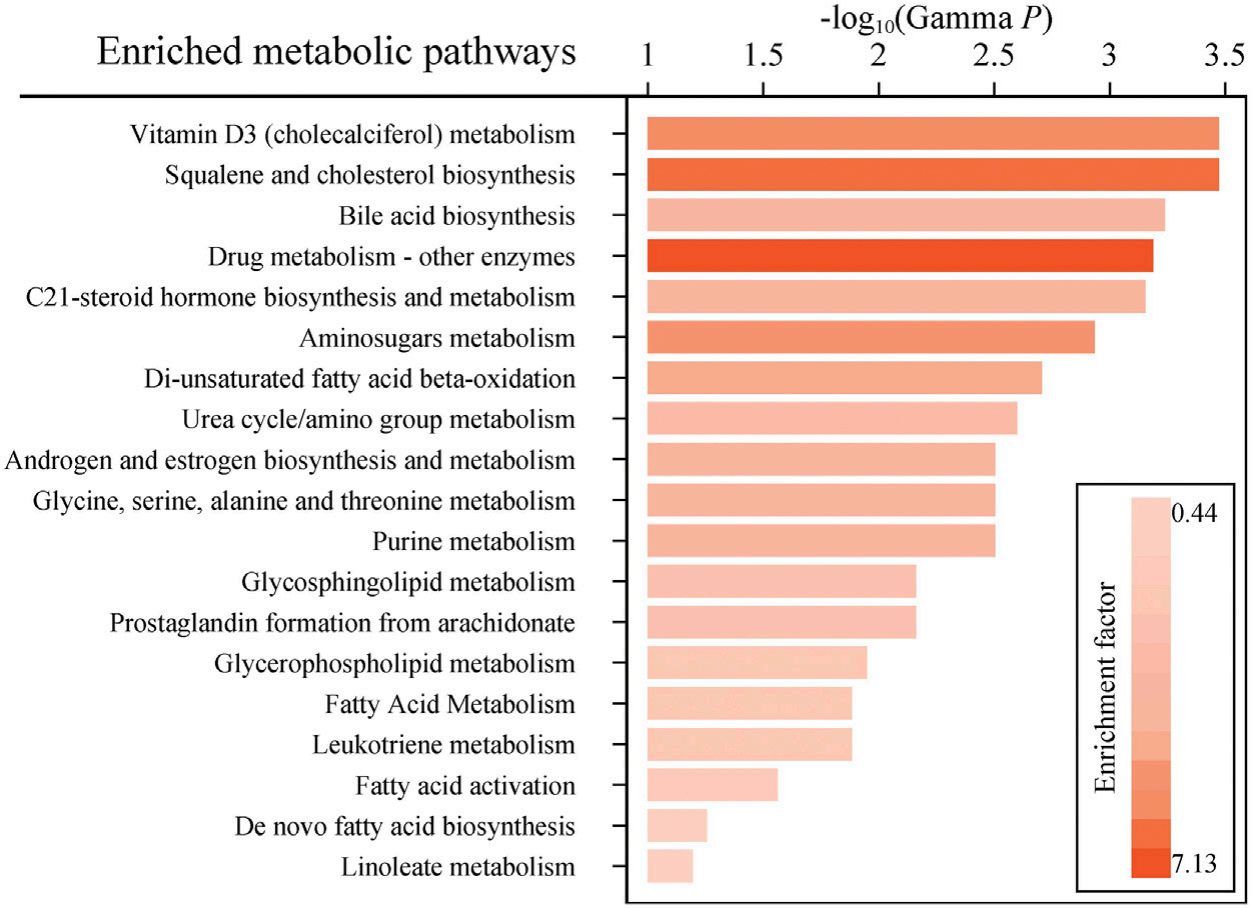
Enriched metabolic pathways predicted by the mummichog algorithm with a *p* value ≤0.05. Colors with gradients of the bars represent relatively high to low values of the enrichment factor.

**FIGURE 4:**
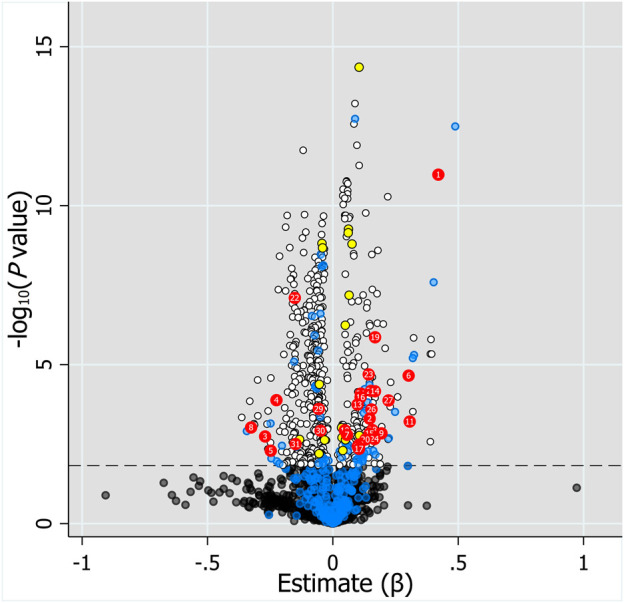
Volcano plot of the association of 2,110 features with MS2 information and log_2_C0/D evaluated by linear mixed model with the fixed effect of each feature and the subjects as the random effect. Horizontal dotted line represents -log10 (*p* value) of 1.823 controlling FDR at 0.05. Black and white dots represent unidentified metabolites with *q* value >0.05 and ≤0.05, respectively. Blue dots below the horizontal dotted line represent identified metabolites with *q* value >0.05, whereas blue dots above the dotted line represent identified metabolites with *q* value ≤0.05 but power <0.8. Red and yellow dots represent endogenous metabolites and drugs or drug metabolites with *q* value ≤0.05 and power ≥0.8, respectively. Labels on dots represent the number of metabolites in Table 2.

Considering the inherent limitation of chemical identification in metabolomics, only features with MS2 information were included in the subsequent analysis. The linear mixed model with the subjects as the random effect was performed in the model development group to evaluate the fixed effect of each feature. The Mummichog algorithm using the m/z and *p* value indicated that the metabolic pathways were significantly enriched in Vitamin D3 (cholecalciferol) metabolism, squalene and cholesterol biosynthesis, bile acid biosynthesis, C21-steroid hormone biosynthesis and metabolism, aminosugars metabolism and so on (Figure 3). The results from the linear mixed model are illustrated as a volcano plot in Figure 4, exhibiting 633 features with significance, of which 112 were identified by accurate m/z and MS2 spectra matching. The power for each identified significant metabolite was evaluated, and the metabolite with a value of power ≥0.80 was retained. Finally, 31 endogenous metabolites were revealed as potential markers of tacrolimus pharmacokinetics (Table 2).

**TABLE 2:** Identified endogenous metabolites with significance using generalized linear mixed model^a^.

Metabolites		RT (min)	Mass (m/z)	Database	Estimate (*β*)	SE	*q* value	SLC
1	Creatinine^b^	0.57	114.0660	HMDB0000562	0.421	0.060	2.482 × 10^-9^	1
2	Indole-3-carbinol^c^	2.06	130.0637	HMDB0005785	0.147	0.042	0.003	1
3	Indole-3-carboxaldehyde^d^	1.2	146.0589	HMDB0029737	−0.270	0.086	0.009	1
4	Carnitine^d^	0.56	162.1126	HMDB0000062	−0.225	0.058	0.001	1
5	Phenylalanine^d^	0.64	166.0855	HMDB0000159	−0.248	0.088	0.021	1
6	Uric acid^b^	0.6	169.0360	HMDB0000289	0.302	0.071	<0.001	1
7	Hippuric acid^c^	1.66	180.0654	HMDB0000714	0.057	0.018	0.008	1
8	Tryptophan^c^	1.2	205.0969	HMDB0000929	−0.326	0.098	0.005	1
9	Indolelactic acid^d^	2.07	206.0798	HMDB0000671	0.194	0.061	0.007	1
10	3-Indoxylsulfate^b^	1.74	212.0029	HMDB0000682	0.048	0.015	0.006	2
11	Pseudouridine^d^	0.6	243.0611	HMDB0000767	0.307	0.089	0.004	1
12	Car 8:0^d^	2.57	288.2178	HMDB0000791	0.106	0.036	0.014	2
13	Testosterone^b^	5.13	289.2155	HMDB0000234	0.099	0.026	0.001	2
14	Car 9:1^d^	2.59	300.2173	CID138309474	0.168	0.042	0.001	2
15	Car 9:0^d^	3.18	302.2317	HMDB0013288	0.146	0.046	0.007	2
16	Car 10:2^d^	2.95	312.2166	CID138251486	0.111	0.028	0.001	2
17	Car 10:1^d^	3.29	314.2335	HMDB0240585	0.104	0.037	0.019	2
18	Car 10:0^c^	3.94	316.248	HMDB0000651	0.099	0.034	0.016	2
19	17 alpha-hydroxyprogesterone^c^	2.43	331.2255	HMDB0000374	0.168	0.035	1.822 × 10^-5^	2
20	Car 12:2^d^	4.23	340.2463	CID138257751	0.130	0.042	0.010	2
21	Car 12:1^d^	4.67	342.2631	CID138234473	0.111	0.028	0.001	2
22	Monoolein^c^	14.5	357.2992	HMDB0011567	−0.152	0.028	1.700 × 10^-6^	2
23	Car 14:2^d^	5.53	368.28	CID138240173	0.143	0.033	<0.001	2
24	Car 14:0^d^	7.29	372.3104	HMDB0005066	0.166	0.054	0.010	2
25	Car 16:2^d^	6.92	396.3098	CID138145981	0.152	0.038	0.001	2
26	Car 18:3^d^	7.53	422.3235	CID138158433	0.154	0.042	0.002	2
27	Car 18:0^d^	10.42	428.3743	HMDB0000848	0.222	0.058	0.001	2
28	Car 20:1^d^	10.72	454.3878	CID138158433	0.158	0.048	0.006	2
29	Taurohyodeoxycholic acid^c^	3.55	464.2837	CID119046	−0.056	0.015	0.002	1
30	Glycocholic acid^c^	4.05	466.3155	HMDB0000138	−0.049	0.015	0.006	1
31	LPC 16:1^d^	8.08	538.3124	HMDB0010383	−0.149	0.050	0.014	2

^a^ Estiamtes (*β*) and standard error (SE) were obtained by linear mixed effects model with each feature as the fixed effect and subject as the random effect on log2C0/D.

^b^ Metabolites with expected effect on dose-adjusted tacrolimus trough concentration (C0/D) supported by clinical study.

^c^ Metabolites with effects on or as the substrates of cytochrome P450 (CYP450) enzymes or transporters involving in tacrolimus pharmacokinetics, supported by animal or cell experiments.

^d^ Metabolites not yet explained. *q* value was calculated by adjusting *p* value using the Benjamini–Hochberg procedure for multiple comparisons. Car, acylcarnitine; LPC, lysophosphatidylcholine; RT, retention time; SLC, Schymanski level of confidence.

### 3.4 Association between concomitant drug use and log_2_C0/D and metabolites

**FIGURE 5:**
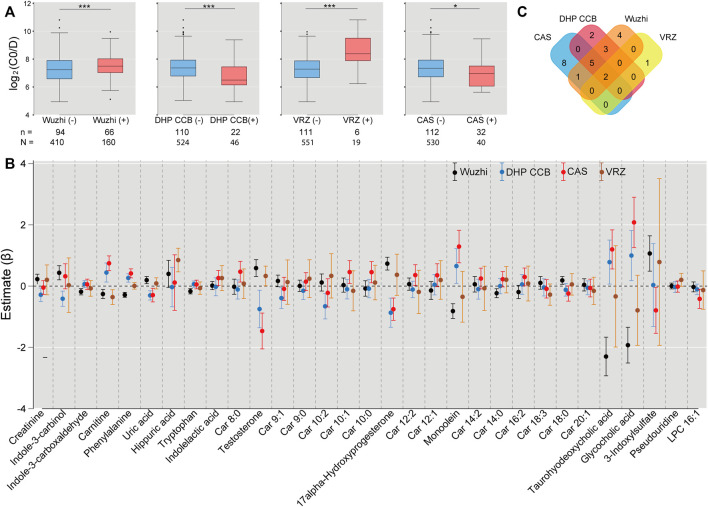
Associations of drug use with log_2_C0/D of tacrolimus and the significant metabolites. **(A)** Wuzhi capsule and voriconazole use exhibited an increased log_2_C0/D, whereas dihydropyridine calcium channel blockers and caspofungin indicated a decreased effect by mixed model analysis. n: number of subjects; N: number of samples; *: *p* < 0.05; ***: *p* < 0.001. **(B)** Comparisons of 31 endogenous metabolites between the samples with and without use of Wuzhi capsule (black), dihydropyridine calcium channel blockers (blue), caspofungin (red), and voriconazole (ochre) by linear mixed model. Each dot represents the estimated coefficient of the metabolites, and the vertical lines with caps represent the 95% confidence interval of estimates. **(C)** Venn diagram of significant metabolites from the comparisons. Wuzhi: Wuzhi capsule; DHP CCB: dihydropyridine calcium channel blockers; VRZ: voriconazole; CAS: caspofungin.

The mixed model analysis exhibited higher log_2_C0/D in the samples taken after voriconazole or Wuzhi capsule use than the samples not related to drug use (voriconazole: *β* = 1.878, SE = 0.203, *p* < 0.001; Wuzhi capsule: *β* = 0.485, SE = 0.085, *p* < 0.001) (Figure 5A), confirming significant pharmacokinetic interactions of voriconazole and Wuzhi capsule with tacrolimus. Conversely, DHP CCB and caspofungin exhibited an inhibitory effect on log_2_C0/D (DHP CCB: *β* = -0.611, SE = 0.130, *p* < 0.001; caspofungin: *β* = -0.309, SE = 0.130, *p* = 0.017). The 31 endogenous metabolites with significance were compared between the samples with and without the four drug uses. The Venn diagram demonstrated two overlapping metabolites, namely, carnitine and testosterone, among all the comparisons and five metabolites, phenylalanine, uric acid, 17alpha-hydroxyprogesterone, monoolein, and taurohyodeoxycholic acid, among the comparisons of Wuzhi capsule, DHP CCB, and caspofungin (Figures 5B,C).

### 3.5 Multiple regression model analysis

**FIGURE 6:**
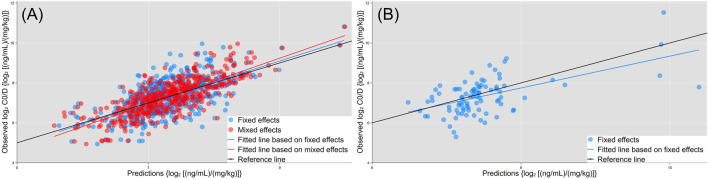
Correlations of observed log_2_C0/D with predictions based on the estimated fixed (blue dots and fitted line) and fixed + random effects (red dots and line) in the model development group. **(A)** Predictions based on the estimated fixed effects (blue dots and fitted line) in the validation group. **(B)** Black lines are the reference line (y = x).

The multivariate model included 11 endogenous metabolites and 12 clinical characteristics as fixed effects (Table 3). A visual inspection of the residual distribution exhibited no obvious deviation from homoscedasticity or normality (Supplementary Figures S3A,B). Figure 6 illustrates the correlations between log_2_C0/D and predictions based on the estimated fixed and fixed plus random effects in the model-developed group (R_fixed_ = 0.64 and R_total_ = 0.78) and predictions based on the estimated fixed effects in the validation group (R_fixed_ = 0.57). The predictive performance of the multiple regression model in the model development and validation groups is presented in Table 4. No significant difference of prediction error (%) between the two groups was found (*β* = 0.871, SE = 1.894, *p* = 0.645) (Supplementary Figure S4).

**TABLE 3:** Parameter estimates of the multiple regression model using LASSO^a^.

Parameters	Estimate	SE	z	*p* Value	95% CI
Endogenous metabolites (a.u.)					
Creatinine	0.328	0.146	2.238	0.025	0.042–0.614
Indole-3-carbinol	−0.108	0.076	−1.422	0.155	−0.257–0.041
Uric acid	0.061	0.126	0.480	0.631	−0.186–0.308
17alpha-Hydroxyprogesterone	−0.080	0.067	−1.198	0.231	−0.211–0.051
Car 12:1	−0.001	0.107	−0.005	0.996	−0.211–0.209
Monoolein	−0.060	0.048	−1.248	0.212	−0.154–0.034
Car 14:2	−0.316	0.179	−1.765	0.078	−0.667–0.035
Car 16:2	0.445	0.190	2.339	0.019	0.073–0.817
Car 18:3	0.123	0.084	1.462	0.144	−0.042–0.288
Car 18:0	−0.058	0.115	−0.506	0.613	−0.283–0.167
Taurohyodeoxycholic acid	0.019	0.028	0.692	0.489	−0.036–0.074
Clinical and basic information					
Days after transplantation (days)	0.001	0.001	1.597	0.110	−0.001–0.003
Cholinesterase (KU/L)	−0.015	0.050	−0.300	0.764	−0.113–0.083
Total protein (g/L)	−0.014	0.010	−1.393	0.164	−0.034–0.006
Globulin (g/L)	0.038	0.016	2.399	0.016	0.007–0.069
Potassium (mM/L)	0.184	0.102	1.792	0.073	−0.016–0.384
Cholesterol (mM/L)	−0.042	0.085	−0.490	0.624	−0.209–0.125
High-density lipoprotein (mM/L)	−0.339	0.206	−1.642	0.101	−0.743–0.065
eGFR (MDRD)	0.0002	0.002	−0.096	0.924	−0.004–0.004
Hemoglobin	0.009	0.003	2.570	0.010	0.003–0.015
Wuzhi capsule use	0.288	0.150	1.918	0.055	−0.006–0.582
Voriconazole use	1.669	0.314	5.323	<0.001	1.054–2.284
DHP CCB use	−0.548	0.203	−2.697	0.007	−0.946–0.15
Intercept	5.959	0.064	92.649	<0.001	5.834–6.084

^a^ Estimates and standard error (SE) were obtained by multiple linear mixed effects model with subject as the random effect on log_2_C0/D using the method of least absolute shrinkage and selection operator (LASSO) augmented with 10-fold cross-validation. Wuzhi capsule, voriconazole, and DHP CCB, use: 0: no use, 1: use. Car, acylcarnitine; DHP CCB: dihydropyridine calcium channel blockers; eGFR: estimated glomerular filtration rate (MDRD).

**TABLE 4:** Predictive performance of the multiple regression model in model development group and validation group.

log_2_C0/D	Model development group		Validation group
	Fixed effects	Fixed + random effects	Fixed effects
ME	−0.010	<0.001	0.148
MAE	0.563	0.450	0.611
MRE	0.077	0.062	0.081
RMSE	0.704	0.575	0.788

ME: mean error; MAE: mean absolute error; MRE: mean relative error; RMSE: root-mean-square error.

## 4 Discussion

The present study applied the method of plasma metabolomics in biomarker finding of tacrolimus pharmacokinetics in liver transplantation recipients. Results of metabolomics identified steady state C0/D of tacrolimus was associated with several endogenous metabolites mainly including medium long chain acylcarnitines, microbiota-derived uremic retention solutes, bile acids, and steroid hormones. Furthermore, levels of several metabolites were obviously altered according to inhibitor or inducer use. A multiple linear mixed model was established and factors including 11 metabolites and clinical information presented a suitable predictive performance for log_2_C0/D.

Our study suggested that biochemistry parameters and metabolites related to renal function, such as creatinine, uric acid, pseudouridine, and eGFR, were associated with tacrolimus log_2_C0/D. Studies have reported creatinine as a factor influencing tacrolimus pharmacokinetics. Fukudo et al. treated serum creatinine as a categorical covariate in adult liver transplant recipients, and tacrolimus clearance was decreased by 19% in subjects with creatinine greater than 1 mg/dl (Fukudo et al., 2003). A linear relation described the serum creatinine effect on clearance, with a coefficient of 0.0801 in adult and pediatric liver transplant recipients (Sam et al., 2006). Pseudouridine and uric acid were also in the list of biomarkers for chronic kidney disease progression using a metabonomic approach (Ye and Mao, 2016; Niewczas et al., 2017).

The observed association between tacrolimus disposition and renal function remains to be explained because tacrolimus undergoes almost no renal elimination (Yu et al., 2018). Studies have demonstrated a twofold higher peak tacrolimus concentration after intraintestinal administration in rats with experimental renal dysfunction than in normal controls (Okabe et al., 2000). The absorption rate of tacrolimus in the intestine might be elevated due to the enhanced nonspecific permeability during renal failure. P-glycoprotein (Pgp) function was suppressed in the liver as opposed to that in the kidney in rats with glycerol-induced acute renal failure, resulting in an increase in tacrolimus bioavailability (Okabe et al., 2002; Huang et al., 2000). Interactions of microbiota-derived uremic retention solutes with drug metabolizing enzymes and transporters were considered to account for that (Prokopienko and Nolin, 2018). Endogenous metabolites associated with tacrolimus log_2_C0/D in the present study also comprised microbiota-derived uremic retention solutes such as hippuric acid, 3-indoxylsulfate, indolelactic acid, and substances involved in the metabolism of such solutes, including indole-3-carboxaldehyde, tryptophan, and phenylalanine. These results confirmed the significance of tryptophan metabolism by intestinal microorganisms into indole and its derivatives, some of which are ligands of the aryl hydrocarbon receptor (AhR) regulating the expression of genes encoding drug metabolizing enzymes and transporters involved in tacrolimus pharmacokinetics (Agus et al., 2018). Although indoxylsulfate is also considered a ligand to AhR, an inhibitory effect on CYP3A was reported in studies by Hubbard et al. and Prokopienko et al. (Hubbard et al., 2015; Prokopienko and Nolin, 2018). These findings are partially concurrent with the findings of the present study. Additionally, indole-3-carbinol, a dietary indole from cruciferous vegetables, has been reported to reverse multidrug resistance to vinblastine and doxorubicin mediated by Pgp (Arora and Shukla, 2003). The inhibitory effect of indole-3-carbinol on Pgp may account for the positive association between indole-3-carbinol and tacrolimus C0/D in the present study.

Glycocholic acid and taurohyodeoxycholic acid are the primary and secondary conjugated bile acids, respectively. Zhang et al. proposed taurohyodeoxycholic acid as a tertiary bile acid because it is derived from redox modification by the host on the steroid skeleton of secondary bile acid, which is produced by microbial enzymes (Zhang et al., 2019). CYP3A4 is the predominant enzyme involved in the transformation (Chen et al., 2014). However, the apparent *Km* for hydroxylation by human liver microsomes was high, resulting in limited levels of taurohyodeoxycholic acid. These levels increased when bile acids accumulated in cholestatic liver disease. Thus, taurohyodeoxycholic acid may be an indicator of CYP3A4 activity and biomarker of tacrolimus pharmacokinetics in liver transplant recipients, as the incidence of biliary complications after liver transplantation was up to 50% (Watt and McCashland, 2008). Collectively, the complex relationships among microbiota-derived uremic retention solutes, metabolites involved in tryptophan metabolism, bile acid metabolism influencing the effects of gut bacteria, and tacrolimus log_2_C0/D indicate the significance of the microbiome on the liver and interorgan communication through the gut-liver-kidney axis proposed in other studies (Patel and Shawcross, 2018; Nigam and Bush, 2019).

Univariate analysis also revealed a positive association between several medium- and long chain acylcarnitines and tacrolimus C0/D. Kim et al. identified five *ω*- or (ω-1)-hydroxylated medium-chain acylcarnitines as novel urinary biomarkers for CYP3A activity in healthy subjects (Kim et al., 2018). The previous study demonstrated that hydroxylated medium-chain acylcarnitine levels in urine correlated positively with tacrolimus clearance. However, a negative correlation was observed in our study. Recently, negative effects of a high-fat diet on intestinal permeability were summarized, and such effects were related to modulation of the expression and distribution of tight junctions, stimulations of a shift to barrier-disrupting hydrophobic bile acids, and induction of oxidative stress and apoptosis in intestinal epithelial cells (Rohr et al., 2020). Tomita et al. evaluated the effects of acylcarnitines on the transporting system in Caco-2 cell monolayers and observed that lauroylcarnitine (acylcarnitine 12:0, Car 12:0) and palmitoylcarnitine (acylcarnitine 16:0, Car 16:0) increased influx and decreased efflux in a manner dependent on their concentration and acyl chain lengths (Tomita et al., 2010). The association of the acylcarnitine profile with tacrolimus C0/D may be attributed to the hydroxylation of medium-chain acylcarnitines by CYP3A4, detergent effects on membranes disrupting membrane barriers, and increasing membrane solubility of long chain acylcarnitines. The exact mechanism of these actions must be further investigated.

Significantly, our results indicated that tacrolimus C0/D increased with days after transplantation in univariate and multivariate regression analysis. Tacrolimus is largely bound to erythrocytes (Woillard et al., 2018), and the increased tacrolimus C0/D with postoperative days in our study may be related to the increase of hematocrit over time after liver transplantation. Additionally, increased bioavailability and the variation of drug-drug interaction could also attribute to that (Riva et al., 2019). In fact, the frequency of comedication with Wuzhi capsule was increased when the graft function was stable in our study.

Multiple regression model established in the study included 11 factors of metabolites, nine factors of clinical biochemistry, and three comedications. There is no significant difference of prediction error (%) between the model development and validation group, indicating a successful application of the model to validation dataset. Inter- and intra-individual variability of tacrolimus pharmacokinetics are relatively large, and most published models performed inadequately in adult liver transplant recipients (Cai et al., 2020). The prediction performance for C0/D (or trough) of the multiple regression model in validation group are relative superior among the published models (Cai et al., 2020). Our findings will also provide an insight into candidate covariates for further model development.

The present study has certain limitations. Firstly, some metabolites exhibiting significance in metabolomics data were not confirmed using authentic standards, which limited the confidence levels of the identification. The identification confidence level is at least two according to the method proposed by Schymanski et al. (Schymanski et al., 2014). The high correlations of typical metabolites between levels obtained in the clinical laboratory and intensity in metabolomics data have confirmed the acceptable identification confidence of these approaches. Secondly, we did not incorporate the genotypic information in recipients and donors, which was suggested to play important roles in tacrolimus pharmacokinetics. The primary objective of the present study is to identify potential plasma metabolomic factors, and metabolomics reflects the comprehensive effects of genetic, environmental, and physiological impacts (Pang et al., 2019). Further studies taking genotypes into account may provide more insights about the interactions between gene and metabolites. Thirdly, the present study is repeated cross-sectional designed, and the limitations inherent to this type of descriptive study do not allow it to establish a causal relationship between metabolic factors and tacrolimus C0/D. Therefore, the ability of the metabolites level prior to the drug administration for predicting of tacrolimus C0/D and guiding tacrolimus dosing should be verified in a cohort study with longitudinal design.

## 5 Conclusion

Thus, the present study focused on metabolomics in liver transplant recipients to identify potential factors associated with tacrolimus dose-adjusted concentration. Microbiota-derived uremic retention solutes, bile acids, steroid hormones, and medium- and long-chain acylcarnitines were the main metabolites identified with significance. Although a multiple regression model incorporating such metabolites was established, further investigation in a larger, more balanced panel is required with additional information on both donor and recipient genotypes and the integration of innovative strategies such as machine learning. Our findings provide valuable insight into metabolomics on individual dosing and guide research on transporters and metabolizing enzymes.

## Data Availability

The original contributions presented in the study are included in the article/Supplementary Material, further inquiries can be directed to the corresponding authors.
