# Supplementary figures and images for: Plasma metabolomic profiling reveals factors associated with dose-adjusted trough concentration of tacrolimus in liver transplant recipients
Source: Front Pharmacol. 2022 Oct 31;13:1045843. doi: 10.3389/fphar.2022.1045843 (PMC9659571; doi:10.3389/fphar.2022.1045843)

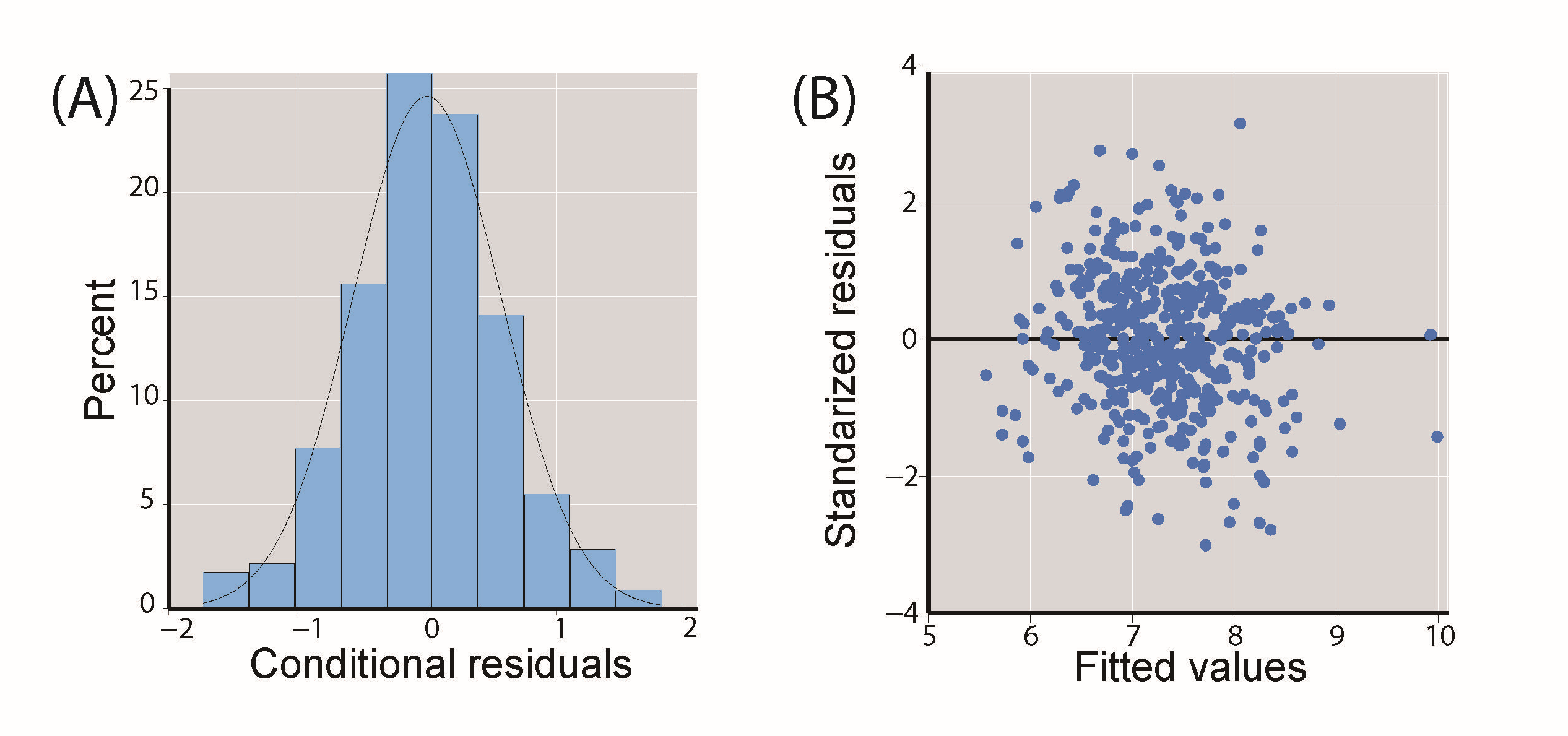

Supplement: Supplementary file 1 [file Image3.TIF]

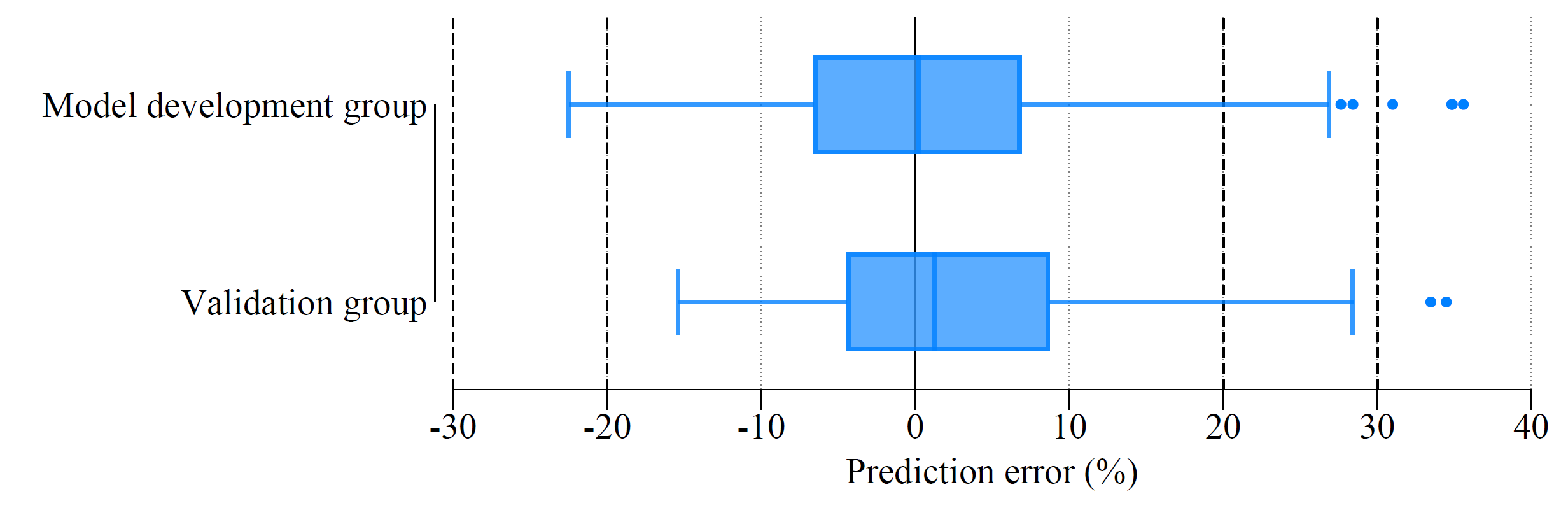

Supplement: Supplementary file 2 [file Image4.TIF]

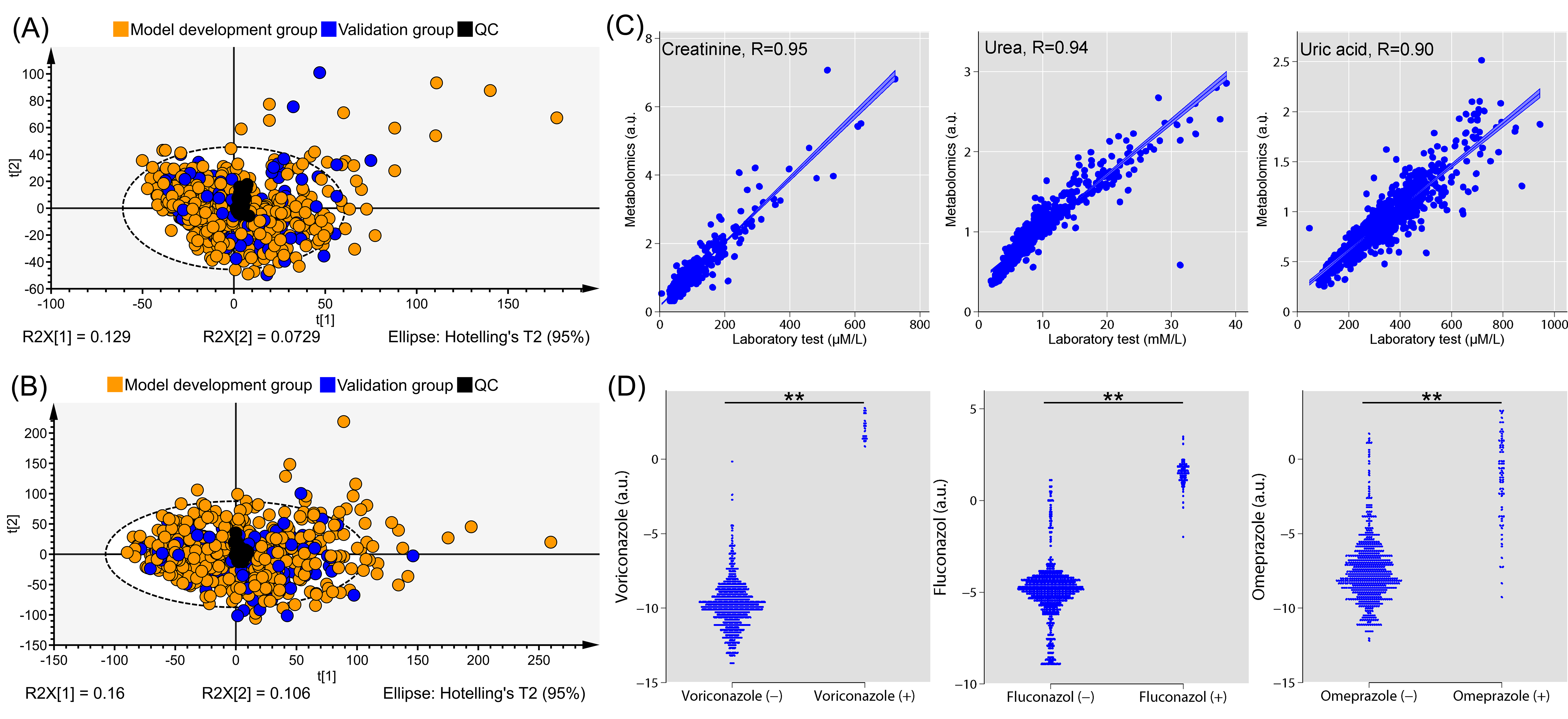

Supplement: Supplementary file 3 [file Image2.TIF]

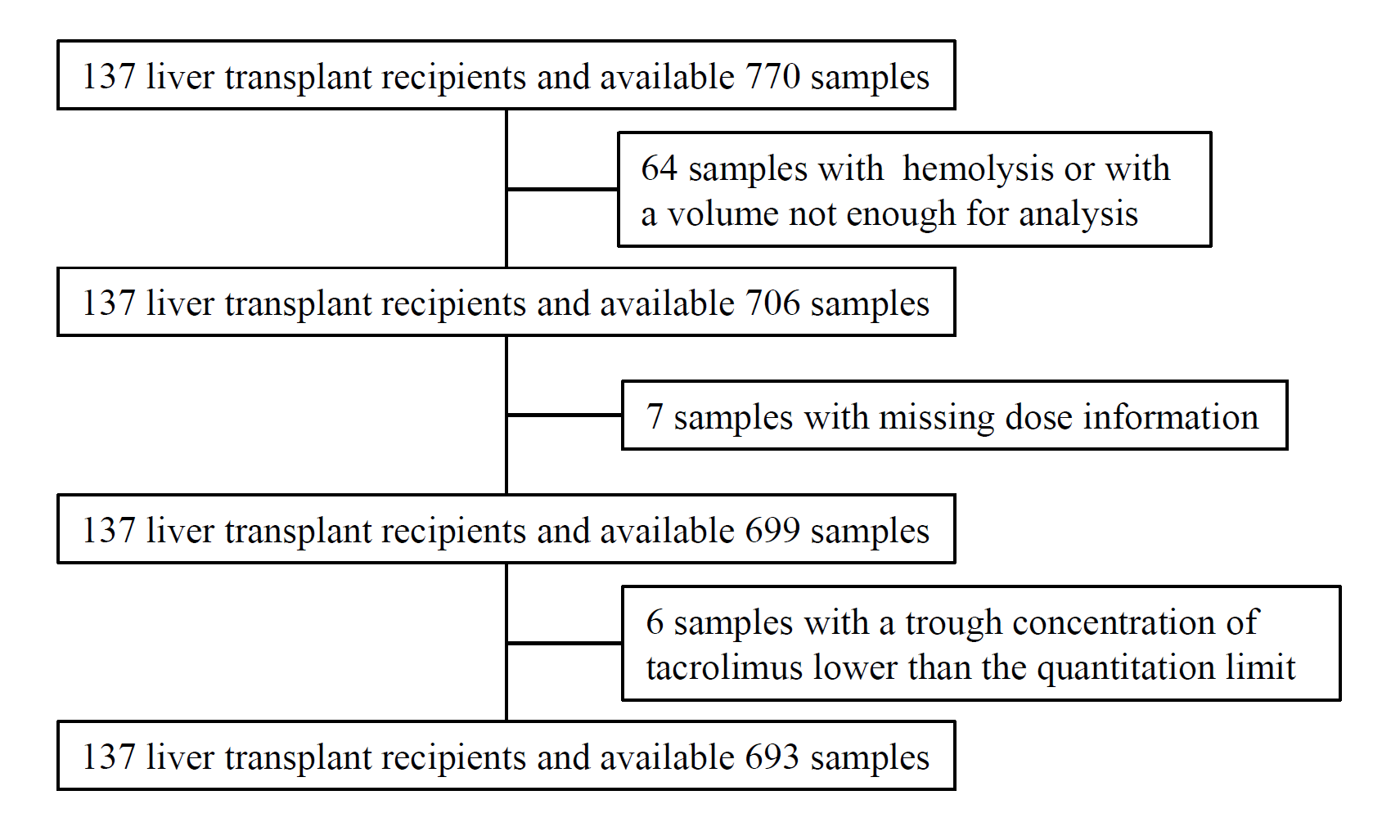

Supplement: Supplementary file 4 [file Image1.TIF]
