# Supplementary material for: Plasma metabolomic profiling reveals factors associated with dose-adjusted trough concentration of tacrolimus in liver transplant recipients
Source: Front Pharmacol. 2022 Oct 31;13:1045843. doi: 10.3389/fphar.2022.1045843 (PMC9659571; doi:10.3389/fphar.2022.1045843)
Supplement: Supplementary file 5 [file DataSheet1.docx]

**Untargeted metabolomics and data analysis**

**Untargeted metabolomics.** Plasma samples were thawed on ice, and a 40-μL aliquot was mixed with 160 μL of cold acetonitrile by vortexing for 5 min. After centrifugation at 20000 g for 15 min at 4°C, the supernatant was collected and centrifuged again. The new supernatant was prepared for injection and separation using ultra-high performance liquid chromatography (UHPLC) (ExionLC AD UHPLC, ABSciex, Framingham, MA, USA) with a Kinetex C18 (2.6 µm, 100 mm × 2.1 mm, Phenomenex, CA, USA) column at 40°C. The mobile phase A comprised water with 0.1% formic acid, whereas the mobile phase B comprised acetonitrile containing 0.1% formic acid. The elution gradient with the flow rate of 0.4 mL/min comprised 10%–30% B over 0-1 min, 30%–95% B over 1-19 min, 95% B over 19-20 min, and 95%–10% B over 20-20.1 min. The system was equilibrated in 90% A for 5 min after each run ending. All samples were analyzed using a TripleTOF 5600+ system (ABSciex, Framingham, MA, USA) in positive and negative electrospray ionization mode. TOF-MS full scan with a mass range of m/z 60-1000 was used to acquire the MS spectra and the MS2 spectra was obtained by information dependent acquisition with the real-time dynamic background subtraction. The acquisition parameters were as follows: ion spray voltage floating, +5500/-4500 V; turbo spray temperature, 550 °C; nebulizer gas (gas 1) pressure, 55 psi; heater gas (gas 2) pressure, 55 psi; curtain gas pressure, 35 psi; declustering potential, ±80 V; The collision energy was set to 35 V with a collision energy spread of 15 V. An automated calibration delivery system infusing the positive/negative calibration solution periodically was adopted to regulate the MS and MS2 data. Pooled plasma sample was prepared as quality control (QC) by mixing equal aliquots of all samples. QC sample replicates were injected after every nine samples sequenced in a random order. QC samples were also repeatedly injected for 10 times before each experiment to stabilize the analytical platform.

**Metabolomics data analysis.** Raw MS data of all the injections including the study samples, QCs, and blanks were converted to the ABF format by using software Reifycs Abf Converter 4.0. Feature detection, spectra deconvolution, peak alignment, and metabolite identification were performed using MS-DIAL v3.4. Features were identified by searching molecular mass data (m/z) and the acquired MS2 spectra against the MassBank database. The m/z tolerance was 0.01 Da and 0.05 Da for MS1 and MS2, respectively, and the cut-off value of the identification score was 80%. Additionally, the Human Metabolome Database (HMDB, http://www.hmdb.ca) and PubChem (<https://pubchem.ncbi.nlm.nih.gov/>) were also used to manually confirm the feature matching. After peak alignment and blank filtering, the results were processed according to the previously reported protocol^1^. Considering the non-normal distribution of most metabolites, log-transformed data were used for subsequent statistical analysis, and the value of intensity was set as +1 for some metabolites below the lower detection limit in several subjects.

REFERENCES

1. Dunn, W. B. *et al.* Procedures for large-scale metabolic profiling of serum and plasma using gas chromatography and liquid chromatography coupled to mass spectrometry. *Nat. Protoc.* **6**, 1060–1083 (2011).
